# Device-assessed physical activity and sedentary behavior in a community-based cohort of older adults

**DOI:** 10.1186/s12889-020-09330-z

**Published:** 2020-08-18

**Authors:** Dori Rosenberg, Rod Walker, Mikael Anne Greenwood-Hickman, John Bellettiere, Yunhua Xiang, KatieRose Richmire, Michael Higgins, David Wing, Eric B. Larson, Paul K. Crane, Andrea Z. LaCroix

**Affiliations:** 1 Kaiser Permanente Washington Health Research Institute, 1730 Minor Ave, Suite 1600, Seattle, WA 98101 USA; 2 University of California, San Diego, 9500 Gilman Dr, La Jolla, CA 92093 USA; 3 University of Washington, 1410 NE Campus Parkway, Seattle, WA 98195 USA

**Keywords:** Sitting time, Exercise, Aging, Accelerometer

## Abstract

**Background:**

Few studies characterize older adult physical activity and sitting patterns using accurate accelerometer and concurrent posture measures. In this descriptive paper, we report accelerometer data collection protocols, consent rates, and physical behavior measures from a population-based cohort study (Adult Changes in Thought, ACT).

**Methods:**

The ACT study holds enrollment steady at approximately 2000 members of Kaiser Permanente Washington aged 65+ without dementia undergoing detailed biennial assessments. In 2016 the ACT-Activity Monitor (ACT-AM) sub-study was initiated to obtain data from wearing activPAL and ActiGraph devices for 7 days following regular biennial visits. We describe the methods protocol of ACT-AM and present characteristics of people who did and did not consent to wear devices. We compute inverse probability of response weights and incorporate these weights in linear regression models to estimate means and 95% confidence intervals (CI) of device-based pattern metrics, adjusted for wear time and demographic factors, and weighted to account for potential selection bias due to device-wear consent.

**Results:**

Among 1885 eligible ACT participants, 56% agreed to wear both devices (mean age 77 years, 56% female, 89% non-Hispanic white, 91% with post-secondary education). On average, those who agreed to wear devices were younger and healthier. Estimated mean (95% CI) activPAL-derived sitting, standing, and stepping times were 10.2 h/day (603–618 min/day), 3.9 h/day (226–239 min/day), and 1.4 h/day (79–84 min/day), respectively. Estimated mean ActiGraph derived sedentary (Vector Magnitude [VM] < =18 counts/15 s), light intensity (VM 19–518 counts/15 s), and moderate-to-vigorous intensity (VM > 518 counts/15 s) physical activity durations were 9.5 h/day (565–577 min/day), 4.5 h/day (267–276 min/day), and 1.0 h/day (59–64 min/day). Participants who were older, had chronic conditions, and were unable to walk a half-mile had higher sedentary time and less physical activity.

**Conclusions:**

Our recruitment rate demonstrates the feasibility of cohort participants to wear two devices that measure sedentary time and physical activity. Data indicate high levels of sitting time in older adults but also high levels of physical activity using cut-points developed for older adults. These data will help researchers test hypotheses related to physical behavior and health in older adults in the future.

## Background

Physical activity is a well-known behavior contributing to cognitive, emotional, functional, and physical health [1]. Most evidence on physical activity and health comes from studies using self-reported measures that in older adults are only moderately correlated with objective measures [2, 3] and that typically overestimate physical activity [4]. With advances in technology, wearable accelerometers can be used to more accurately measure physical activity in a relatively low burden manner for participants [5]. Many epidemiologic studies now use accelerometry including the Women’s Health Initiative [6], the Women’s Health Study [7], and the British Regional Heart Study [8].

In addition to physical activity, growing evidence also suggests the importance of (minimizing) sedentary time in healthy aging [1, 9]. Sedentary time consists of behaviors performed while sitting or lying down at low levels of energy expenditure; sleep and standing without ambulation are not considered sedentary behaviors [10]. Common sedentary behaviors include watching television, riding in a car, or working on a computer. Population levels of sedentary time are high [11], and older adults are estimated to have the highest levels of all age groups [11, 12]. Sedentary behavior has been shown to adversely affect health among older adults [13, 14].

Prior epidemiologic data has relied on self-reported assessments of sedentary behaviors [15] while newer studies include data from waist-worn accelerometers [6]. Self-reported sedentary behaviors do not correlate strongly with waist-worn accelerometer measures in adult and older adult populations and tend to underestimate sedentary time by as much as 5-h/day [2] on average [2, 13, 16, 17]. Accurately recalling and reporting patterns of sitting, standing, and small non-exercise based activity that can occur throughout the day is very cognitively challenging for older adults [18, 19]. Waist-worn accelerometers, however, also encounter challenges in measuring sedentary behaviors because they can misclassify standing time as sedentary time and can misclassify time in an automobile as active time, making accurate population estimates of sitting and lying time and their associations with health difficult [20].

The Adult Changes in Thought (ACT) Study is an on-going longitudinal cohort study that began in 1994 to investigate risk factors for development of dementia and has since provided unique opportunity to study factors of healthy aging more broadly. Self-reported exercise has been measured since the cohort’s inception. In a prior publication, self-report of exercising at least 3 times per week for at least 15 min was associated with a lower risk for incident dementia [21]. A limitation of the cohort has been the lack of device-based assessments of physical activity and sedentary time, thus prohibiting the study’s ability to measure detailed patterns of movement, inactivity, and sitting time and to examine their potential impact on aging-related outcomes.

Starting in 2016, we added activPAL and ActiGraph monitors to more accurately capture the spectrum of sedentary and physically active patterns among older men and women (ACT Activity Monitor, ACT-AM, sub-study). This report describes the data collection procedures for these new devices, consent rates, and presents a description of the objectively observed patterns of physical activity and sedentary behavior measured within the ACT-AM cohort.

## Methods

### The ACT parent study

The ACT prospective cohort study began in 1994 by enrolling adults aged 65+ without dementia who had been randomly sampled from the King County membership panels of Kaiser Permanente Washington (KPWA) (formerly Group Health), an integrated health care delivery system in the state of Washington. Study participants undergo biennial follow-up visits to screen for incident dementia. Those with low cognitive screening scores at biennial visits undergo additional diagnostic workup and are brought to a consensus conference to determine whether dementia is present per DSM-IV criteria and to identify subtype. Participants are followed until earliest of dementia onset, study disenrollment, or death. Starting in 2000 an expansion cohort was recruited to increase the study size, and in 2005 continuous enrollment of participants began to ensure a stable active cohort of approximately 2000 older adults. Study visits are completed at a central research clinic or at the participant’s home, based on participant preference [22]. For the original cohort enrolled 1994–1996, home visits were requested by about 15% at study enrollment, while they were requested for upwards of 85% of surviving members of that cohort at the most recent biennial study cycle.

Well established procedures [23] during biennial visits include cognitive testing, physical performance testing, and collection of a variety of self-reported health and activity measures. Additionally, because ACT participants are drawn from KPWA, the study collects additional data elements from participants’ electronic health records (EHR) and other KPWA automated data systems that include elements such as pharmacy prescription fills, diagnoses and procedures, and laboratory measures taken as part of clinical care. There are sub-studies which collect chart review, genetic, neuroimaging, and cerebrospinal fluid data as well as brain autopsy measures. All procedures are approved by the Kaiser Permanente Washington (KPWA) institutional review board.

### ACT-AM sub-study enrollment

At clinic-based and home-based biennial visits beginning in April 2016, participants were consented to wear activity monitoring devices. If participants were wheelchair bound, receiving hospice or care for a critical illness, residing in a nursing home, or if memory problems became evident during testing, they were not eligible to participate in the activity monitor sub-study. Upon obtaining consent, a research staff member explained how to wear the activity monitors and provided participants with a 7-day log in which they were asked to detail device wear and sleep. Extra supplies and photographs for properly securing the devices were provided. Participants were also provided with a take-home questionnaire which included measures of self-reported sedentary behavior and physical activity. A pre-addressed, pre-paid, padded envelope was provided to mail devices, wear/sleep logs, and the questionnaire back to the study team upon completion of the 7-day wear period.

### ACT-AM devices and measurements

Two research grade accelerometers were deployed—the waist-worn Actigraph wGT3X+ and the thigh-worn activPAL micro. Participants were given the option to consent to wear one or both devices, or decline both. Those who consented were asked to wear their device(s) for 7 calendar days. Both devices were secured so that they did not have to be removed and participants were encouraged to wear them for 24 h per day with the exception of swimming, bathing or showering for the ActiGraph. During the wear period, participants were asked to keep a log noting the times they went to bed and the time they got out of bed (collectively used to calculate time in bed), along with notes regarding any removal of the waist-worn device for reasons other than bathing or showering. However, if the log was a barrier to participation, participants were not required to complete one. Wear and sleep logs were double entered into a database that was quality checked for completeness and accuracy. When data were missing, the mean of the in-bed and awake time from the person’s existing in-bed or out-of-bed times were used. When all in-bed or out-of-bed times were missing (i.e. participant did not complete or did not return a log) the average in-bed and/or out-of-bed times for the cohort were used (*N* = 37).

The ActiGraph wGT3X+ (ActiGraph LLC, Pensacola, FL, USA) is a well-validated accelerometer, particularly for measuring light, moderate, and vigorous intensity physical activity [3, 24, 25]. The device is small, lightweight, and worn on an elastic belt secured around the waist so the device rests on the right side at the level of the suprailiac crest. Devices were initialized using ActiLife software (v 6.13.3) to capture data at 30 Hz. Upon return, staff downloaded raw data (i.e. 30 Hz data presented in gravitational units) and processed these into a proprietary counts variable at 15 s epochs using the normal filter. The algorithm developed by Choi et al. (2011) [26] was used to assess wear compliance with a definition of non-wear time as periods of at least 90 consecutive minutes of zero counts per minute (cpm) using vector magnitude. We identified in-bed time using information from sleep logs. An adherent day was defined as any day with ≥10 h of awake wear time. Daily summaries were averaged over all adherent days to generate person-level data for analysis. Participants were included for analysis of physical activity if they had ≥4 adherent days. Calibrated cut-points developed in a Women’s Health Initiative laboratory study were applied to the Actigraph data to more appropriately represent older adult physical activity [25]. Specifically, intensity classifications using vector magnitude counts per 15 s epoch were as follows: ≤18 for sedentary time, 19–518 for light-intensity physical activity (LPA), and > 518 for moderate-to-vigorous physical activity (MVPA) [25].

The activPAL micro (PAL Technologies, Glasgow, Scotland, UK) is very small and lightweight; it was secured to the front center of the thigh using a medical grade adhesive tape (Tegaderm). The device was packaged in a waterproof casing so that it does not have to be removed for bathing, showering, or swimming to improve compliance [27]. activPAL is currently considered the most accurate field-based measure of sitting time and sit-to-stand transitions [28–31]. The device has been used in older adults, particularly in intervention studies [32], and it is also being used in the Seniors USP (Understanding Sedentary Patterns) study based in Scotland [27], the Maastricht Study [33], and the iDATA study [34]. Data were converted to event-level files using proprietary PAL Technologies software. The events files were then processed by first collapsing consecutive activities of the same activity type, then adjusting the cumulative steps accordingly. In-bed time determined using the sleep log was removed from the data using batch processing programs in R. Heatmaps that depict sitting/lying, standing, stepping and in-bed time were created to visualize the activPAL data and were inspected to check for anomalies suggestive of potential invalid wear or device malfunction (e.g., only sitting was recorded), with corrections or exclusions made, as needed. As with the ActiGraph data, we only included days with ≥10 h of awake (out-of-bed) wear time and averaged the daily summaries across these days to generate person-level data for analysis. To be included in analysis of sedentary behaviors, participants were required to have at least 4 days of valid data.

#### Covariates

ACT study data collected at each biennial visit and included in this manuscript were: demographics such as age, sex, race/ethnicity, and education; measured body mass index (BMI); self-reported history of comorbidities including hypertension, coronary artery disease (myocardial infarction, coronary artery bypass grafting, coronary angioplasty, or angina), and cerebrovascular disease (stroke, transient ischemic attack, or carotid endarterectomy); self-rated health and whether walking was regularly engaged in for exercise; self-reported limitations in activities of daily living and level of difficulty with walking a half-mile; and measures of depressive symptoms and cognitive functioning. Depressive symptoms were measured using the Center for Epidemiologic Studies Depression Scale (CES-D) and presence of significant depressive symptoms was classified as scores of 10 or higher [35]. Cognitive function was assessed using the Cognitive Abilities Screening Instrument (CASI) [36] with presence of cognitive impairment defined as a score < 86 and/or referral for additional diagnostic workup.

#### Analysis

First, we summarized information on the total number of ACT participants who consented to device-wear and how many ultimately provided sufficient valid wear for inclusion in analyses of the device data. Next, we compared participant characteristics of those that did and did not consent to wear each device. In order to account for potential selection bias due to factors related to device-wear consent, we then estimated a logistic regression model for the binary outcome of consent as a function of several demographic, behavioral, and health-related covariates. Separate models were estimated for ActiGraph and activPAL consent. We used predictions from these models to construct inverse probability of response (consent) weights which were then incorporated in all estimates of the ActiGraph and activPAL device-based measures [37, 38]. The goal of the weighting was to yield estimates of device-based activity and sitting pattern data that are generalizable to the broader ACT cohort, which, as described above, is based on a random sample of age-eligible health plan enrollees and is representative of the population of people over age 65 who get care at Kaiser Permanente Washington. Linear regression was used to provide estimated means (and 95% confidence intervals) of each average daily device-based pattern metric overall and by participant characteristics, adjusted for wear time and demographic factors (age, gender, race/ethnicity, and education) and weighted to account for selection. Models were estimated using weighted generalized estimating equations with standard errors estimated via the robust sandwich estimator [39]. Data processing, modeling, and summarizing used a combination of R, version 3.5.2 (R Foundation for Statistical Computing, Vienna, Austria), and SAS software, version 9.4 (SAS Institute, Inc., Cary, NC).

## Results

### Participant characteristics

**Fig. 1:**
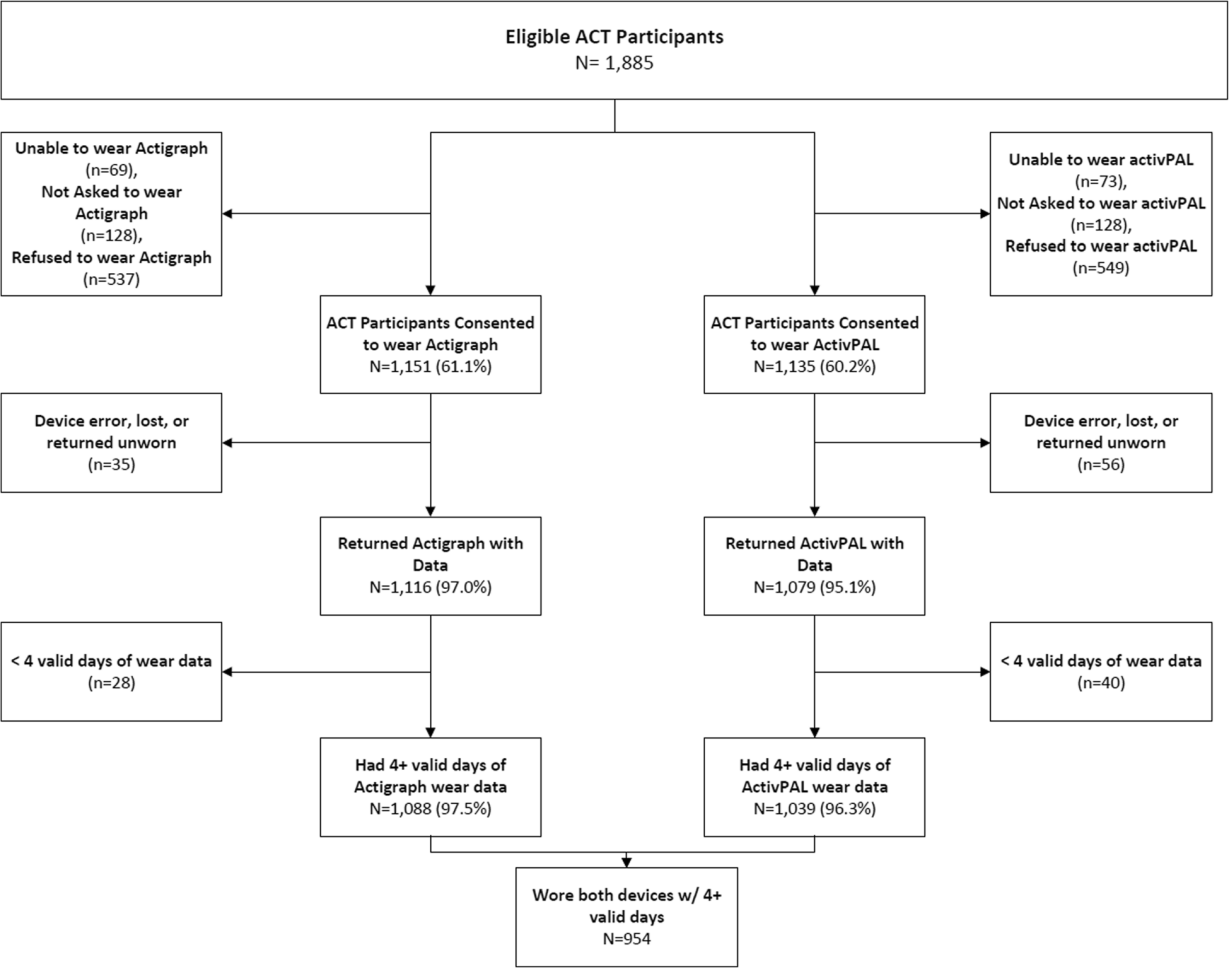
Flow Diagram for the ACT Activity Monitoring (ACT-AM) Sub-Study

Device data collection began in April 2016 and was completed in July 2018. Among 1885 ACT participants initially eligible during this period, 1151 (61%) consented to wear Actigraph and 1135 (60%) consented to wear activPAL;1054 (56%) consented to wear both. The most common reasons for non-consent included: not interested (33% ActiGraph, 28% activPAL), poor health or illness (18% ActiGraph, 17% activPAL), skin issues or discomfort (5% ActiGraph; 8% activPAL), and cognitive impairment (9% ActiGraph, 8% activPAL). Reasons for declining were unknown for 9% of those that refused ActiGraph and 8% of those who refused activPAL. Among participants that wore devices, 1088 (95%) Actigraph wearers and 1039 (92%) activPAL wearers had at least 4 days of valid data. Figure 1 depicts the participation cascade for the cohort.

Table 1 presents sample demographics by consent status. Overall, those consenting to wear devices were generally younger and healthier than those who did not consent. Of note, approximately 21% of participants who did not consent to wear devices were age 90+, while only approximately 6% of consenting participants were in this age category. Participants who consented more commonly reported excellent or very good health (61%) compared to those who did not consent (40%) and also had lower prevalence of comorbid chronic conditions (hypertension, cerebrovascular disease, etc.). Further, participants who consented to device-wear were more likely to self-report regularly walking for exercise compared to those who did not consent (approximately 54% vs. 40%).

**Table 1:** Comparison of participant characteristics between those who did and did not consent to wear device

	ActiGraph		activPAL	
	Consent(***N*** = 1151)	Non-consent(***N*** = 734)	Consent(***N*** = 1135)	Non-consent(***N*** = 750)
	%	%	%	%
Age category				
65–69	13	5	12	6
70–74	30	17	30	18
75–79	23	19	23	20
80–84	18	19	18	19
85–89	11	18	11	17
90+	6	22	6	21
Male	44	39	44	40
Non-Hispanic white	88	87	89	87
Post-secondary education	90	85	91	85
BMI				
Underweight	1	2	1	2
Normal	37	39	37	38
Overweight	39	35	39	35
Obese	23	25	23	25
Self-rated health				
Excellent	19	9	19	10
Very good	42	31	42	31
Good	31	41	31	40
Fair	7	15	7	15
Poor	1	4	1	4
# of ADLs with difficulty				
0	79	63	77	65
1	15	19	16	17
2+	6	18	7	17
Significant depressive symptoms (CESD score ≥ 10)	9	12	9	11
Cognitive impairment	3	15	3	15
Regularly walk for exercise	54	39	53	41
Difficulty walking half mile				
None	75	53	74	55
Some	15	18	15	17
A lot / Unable	11	29	11	28
Hypertension	48	57	48	57
Coronary artery disease	13	20	13	19
Cerebrovascular disease	10	16	10	15

Column percentages are shown among non-missing

### activPAL data patterns

Table 2 displays mean activPAL-derived activity metrics across various sociodemographic and health factors in our sample. Overall, estimated mean sitting, standing, and stepping times were 10.2 h/day (610 min/day; 95% CI: 603–618 min/day), 3.9 h/day (233 min/day; 95% CI: 226–239 min/day), and 1.4 h/day (81 min/day; 95% CI: 79–84 min/day), respectively, with a mean daily step count of 6302 steps/day (95% CI: 6108–6496). Further, people were estimated to have an average of 43 sit-to-stand transitions/day (CI: 42–44) with a mean daily sitting bout duration of 17 min/bout (CI: 16–18). Adjusting for wear time and demographics, older age tended to be associated with higher levels of sitting time (*p* < 0.001), longer sitting bout duration (*p* = 0.011), and fewer daily steps (*p* < 0.001). For example, people aged 90+, on average, sat for 11.6 h/day (696 min/day; 95% CI: 667–726 min/day), had a mean sitting bout duration of 25 min/bout (95% CI: 15–35), and took 3553 daily steps (95% CI: 2934–4172); whereas, people under 70 sat for an average of 9.8 h/day (585 min/day; 95% CI: 565–605 min/day), had a mean sitting bout duration of 15 min/bout (95% CI: 14–16), and took 8102 daily steps (95% CI: 7517–8687). Men exhibited higher levels of sitting time than women (*p* < 0.001) and modestly longer sitting bout durations (*p* = 0.012) but no significant difference in step count (*p* = 0.766). The only significant differences observed by race/ethnicity were for time spent stepping (*p* = 0.007) and step count (*p* = 0.017). BMI was also associated with the activPAL sedentary and activity pattern metrics (all *p* < 0.001), with obese individuals exhibiting levels of sitting (11.1 h/day or 664 min/day; 95% CI: 648–679 min/day) nearly an hour and a half greater than those with normal weight (9.7 h/day or 581 min/day; 95% CI: 570–592 min/day) and having 8 fewer sit-to-stand transitions per day and accumulating nearly 3000 fewer daily steps. We observed no associations between education or cognition and any of the pattern metrics. Reported difficulties with walking a half-mile was strongly associated with step counts, transitions, sitting bout durations, and total time spent sitting, standing, or stepping (all *p* ≤ 0.001). People reporting no difficulty had an average daily step count (7224 steps/day; 95% CI: 6995–7453) nearly twice that of those reporting a lot of difficulty or inability (3668 steps/day; 95% CI: 3292–4044) and had notably lower levels of sitting time (9.8 vs. 11.5 h/day) and shorter sitting bout durations (15 vs. 23 min/bout). Comorbidities such as hypertension and coronary artery disease, as well significant depressive symptoms, also tended to be associated with lower levels of activity as measured by higher sitting time (all *p* < 0.05) and fewer steps (all *p* ≤ 0.001).

**Table 2:** Patterns of sedentary behavior and physical activity measured by activPAL^a^

	N	Sitting time (mins/day)	Standing time (mins/day)	Stepping time (mins/day)	Steps (steps/day)	Sit-to-Stand transitions (number/day)	Sitting bout duration (mins/bout)	Sitting bouts of 30+ mins (bouts/day)
Overall	997	610 (603, 618)	233 (226, 239)	81 (79, 84)	6302 (6108, 6496)	43 (42, 44)	17 (16, 18)	5.9 (5.8, 6.0)
Age category								
65–69	126	585 (565, 605)	238 (223, 254)	101 (94, 107)	8102 (7517, 8687)	44 (42, 46)	15 (14, 16)	5.6 (5.3, 5.9)
70–74	300	583 (570, 596)	244 (234, 255)	96 (92, 101)	7638 (7216, 8060)	45 (43, 46)	15 (14, 16)	5.6 (5.4, 5.8)
75–79	235	606 (590, 621)	234 (222, 247)	84 (79, 89)	6492 (6069, 6915)	42 (40, 44)	17 (15, 18)	5.8 (5.6, 6.1)
80–84	173	608 (590, 627)	239 (224, 254)	76 (72, 81)	5752 (5346, 6159)	42 (41, 44)	16 (15, 18)	5.9 (5.6, 6.1)
85–89	114	625 (602, 647)	235 (214, 255)	64 (59, 70)	4852 (4410, 5294)	42 (40, 45)	18 (16, 20)	6.0 (5.7, 6.3)
90+	49	696 (667, 726)	179 (155, 203)	49 (41, 56)	3553 (2934, 4172)	41 (36, 46)	25 (15, 35)	7.0 (6.4, 7.5)
*p*-value		< 0.001	< 0.001	< 0.001	< 0.001	0.138	0.011	< 0.001
Gender								
Female	552	593 (583, 604)	249 (240, 258)	81 (79, 84)	6275 (6034, 6516)	44 (43, 46)	15 (15, 16)	5.7 (5.5, 5.8)
Male	445	631 (621, 642)	212 (203, 220)	81 (78, 85)	6336 (6015, 6657)	41 (40, 43)	19 (16, 22)	6.2 (6.0, 6.3)
*p*-value		< 0.001	< 0.001	0.889	0.766	0.002	0.012	< 0.001
Race/ethnicity								
People of color	106	607 (583, 631)	244 (223, 265)	73 (67, 80)	5655 (5080, 6230)	44 (40, 47)	17 (15, 19)	5.6 (5.3, 5.9)
Non-Hispanic white	891	610 (603, 618)	231 (225, 238)	82 (80, 85)	6393 (6189, 6598)	43 (42, 44)	17 (16, 18)	5.9 (5.8, 6.1)
*p*-value		0.797	0.263	0.007	0.017	0.579	0.735	0.078
Education								
High school or less	90	614 (585, 644)	228 (204, 252)	82 (75, 89)	6347 (5732, 6963)	42 (38, 46)	17 (13, 20)	6.0 (5.6, 6.4)
Post-secondary	907	609 (602, 617)	233 (227, 240)	81 (79, 84)	6296 (6097, 6495)	43 (42, 44)	17 (16, 19)	5.9 (5.8, 6.0)
*p*-value		0.746	0.663	0.879	0.874	0.735	0.778	0.597
BMI								
Underweight	9	619 (500, 737)	226 (127, 325)	79 (57, 102)	6041 (4545, 7537)	47 (35, 60)	15 (11, 20)	6.4 (4.1, 8.8)
Normal weight	366	581 (570, 592)	251 (242, 260)	92 (88, 95)	7266 (6930, 7602)	46 (44, 48)	16 (13, 18)	5.5 (5.3, 5.7)
Overweight	393	604 (593, 616)	235 (226, 245)	84 (81, 88)	6497 (6203, 6790)	43 (42, 44)	16 (15, 17)	5.8 (5.7, 6.0)
Obese	229	664 (648, 679)	199 (186, 213)	61 (57, 65)	4512 (4201, 4822)	38 (37, 40)	21 (19, 22)	6.6 (6.4, 6.8)
*p*-value		< 0.001	< 0.001	< 0.001	< 0.001	< 0.001	< 0.001	< 0.001
Self-rated health								
Excellent	183	570 (555, 585)	257 (245, 270)	97 (92, 102)	7683 (7199, 8167)	45 (43, 46)	14 (14, 15)	5.3 (5.1, 5.5)
Very good	432	600 (591, 610)	237 (229, 245)	87 (84, 90)	6826 (6538, 7114)	43 (42, 44)	16 (15, 17)	5.8 (5.7, 6.0)
Good	304	623 (609, 636)	226 (215, 237)	76 (72, 80)	5730 (5398, 6061)	43 (42, 45)	17 (16, 19)	6.1 (5.9, 6.3)
Fair	68	666 (636, 696)	199 (172, 225)	59 (52, 66)	4495 (3935, 5054)	40 (36, 43)	24 (16, 31)	6.1 (5.6, 6.7)
Poor	10	604 (524, 685)	260 (179, 340)	60 (45, 74)	4337 (3175, 5500)	41 (32, 50)	15 (11, 19)	6.1 (5.1, 7.1)
*p*-value		< 0.001	< 0.001	< 0.001	< 0.001	0.207	< 0.001	< 0.001
Depressive symptoms								
CESD score < 10	908	607 (599, 615)	234 (227, 240)	83 (81, 86)	6467 (6263, 6671)	43 (42, 44)	17 (15, 18)	5.9 (5.7, 6.0)
CESD score ≥ 10	89	638 (608, 667)	223 (196, 249)	64 (57, 70)	4805 (4296, 5313)	40 (37, 43)	20 (17, 22)	6.2 (5.9, 6.6)
*p*-value		0.049	0.430	< 0.001	< 0.001	0.035	0.093	0.063
Cognitive impairment								
No	976	609 (602, 616)	233 (227, 239)	82 (80, 84)	6335 (6139, 6531)	43 (42, 44)	16 (16, 17)	5.9 (5.8, 6.0)
Yes	21	625 (570, 680)	224 (180, 268)	75 (60, 89)	5742 (4664, 6821)	41 (31, 51)	30 (13, 47)	5.3 (4.4, 6.2)
*p*-value		0.582	0.701	0.347	0.291	0.642	0.109	0.165
Difficulty walking half mile								
None	751	587 (579, 595)	245 (239, 252)	91 (89, 94)	7224 (6995, 7453)	44 (43, 45)	15 (14, 16)	5.7 (5.5, 5.8)
Some	141	624 (605, 643)	231 (215, 247)	69 (63, 74)	5081 (4640, 5522)	41 (38, 44)	19 (16, 21)	6.1 (5.8, 6.4)
A lot / Unable	105	691 (668, 714)	182 (162, 202)	51 (46, 56)	3668 (3292, 4044)	39 (36, 42)	23 (19, 28)	6.6 (6.3, 7.0)
*p*-value		< 0.001	< 0.001	< 0.001	< 0.001	0.001	< 0.001	< 0.001
Hypertension								
No	536	595 (585, 605)	244 (235, 252)	85 (82, 88)	6635 (6363, 6906)	44 (42, 45)	16 (15, 17)	5.7 (5.6, 5.9)
Yes	461	625 (614, 636)	222 (213, 230)	77 (74, 81)	5978 (5707, 6248)	42 (41, 44)	18 (16, 20)	6.0 (5.9, 6.2)
*p*-value		< 0.001	< 0.001	< 0.001	0.001	0.257	0.008	0.005
Coronary artery disease								
No	869	605 (597, 614)	235 (229, 242)	83 (81, 86)	6465 (6247, 6682)	43 (42, 44)	17 (15, 19)	5.8 (5.7, 6.0)
Yes	128	635 (613, 657)	217 (198, 236)	71 (66, 77)	5407 (4935, 5880)	41 (39, 44)	17 (14, 20)	6.3 (6.0, 6.6)
*p*-value		0.015	0.081	< 0.001	< 0.001	0.156	0.868	< 0.007

^a^Values shown in the table are the means (95% confidence intervals) of the average daily pattern metric adjusted for wear time and demographic factors (age, gender, race/ethnicity, and education) and weighted to account for selection due to factors related to device-wear consent. *P*-values correspond to tests for differences in means across levels of the factors. The selection model used to generate weights was based on all covariates shown in Table 1. activPAL-wear participants missing covariates that were used in the selection model (*N* = 42 participants) were excluded from the above weighted summary table

### ActiGraph data patterns

Table 3 displays mean ActiGraph-derived activity metrics across various sociodemographic and health factors in our sample. Estimated mean sedentary time, overall, was 9.5 h/day (571 min/day; 95% CI: 565–577 min/day), while mean time spent in total LPA and MVPA were 4.5 h/day (272 min/day; 95% CI: 267–276 min/day) and 1.0 h/day (61 min/day; 95% CI: 59–64 min/day), respectively. Patterns observed across demographic and health characteristics were consistent with those found with the activPAL data. For example, older age, higher BMI, and greater difficulty walking a half mile were all associated with higher levels of sedentary time and lower levels of MVPA (all *p* < 0.001) based on wear time and demographic adjusted models. Men had higher levels of sedentary time (10.0 h/day or 597 min/day; 95% CI: 589–605 min/day) and lower levels of LPA (4.1 h/day 247 min/day; 95% CI: 241–254 min/day) than women (sedentary 9.2 h/day or 551 min/day; 95% CI: 544–559 min/day; LPA 4.8 h/day or 290 min/day; 95% CI: 284–296 min/day), but they did not differ in levels of MVPA (*p* = 0.305) with both genders spending approximately an hour per day doing MVPA. Race/ethnicity was not associated with sedentary time or LPA but was associated with MVPA (*p* = 0.002), with people of color having 10 min less MVPA per day, on average, than Non-Hispanic white people. Lastly, as with activPAL, education and cognition were not associated with the ActiGraph metrics, but comorbidities were; people with hypertension, coronary artery disease, or significant depressive symptoms tended to exhibit approximately 20 to 30 min more sedentary time and about 15 min less MVPA than people without.

**Table 3:** Patterns of sedentary behavior and physical activity measured by ActiGraph^a^

	N	Sedentary time (mins/day)^b^	LPA time (mins/day)^b^	MVPA time (mins/day)^b^
Overall	1044	571 (565, 577)	272 (267, 276)	61 (59, 64)
Age category				
65–69	129	547 (532, 562)	270 (258, 281)	87 (81, 94)
70–74	317	547 (537, 557)	276 (268, 283)	81 (77, 86)
75–79	250	566 (554, 579)	271 (261, 280)	67 (62, 71)
80–84	185	567 (553, 580)	285 (275, 296)	52 (47, 58)
85–89	112	605 (586, 624)	263 (246, 279)	36 (31, 41)
90+	51	628 (607, 649)	253 (234, 272)	23 (18, 28)
*p*-value		< 0.001	0.027	< 0.001
Gender				
Female	582	551 (544, 559)	290 (284, 296)	62 (59, 65)
Male	462	597 (589, 605)	247 (241, 254)	60 (57, 63)
*p*-value		< 0.001	< 0.001	0.305
Race/ethnicity				
People of color	118	576 (559, 594)	275 (260, 290)	53 (47, 58)
Non-Hispanic white	926	570 (564, 576)	271 (266, 276)	63 (60, 65)
*p*-value		0.522	0.636	0.002
Education				
High school or less	100	571 (547, 595)	269 (250, 289)	63 (57, 70)
Post-secondary	944	571 (565, 577)	272 (267, 277)	61 (59, 63)
*p*-value		0.984	0.816	0.532
BMI				
Underweight	10	521 (417, 625)	317 (226, 407)	66 (46, 86)
Normal weight	388	535 (527, 543)	297 (290, 304)	72 (68, 75)
Overweight	414	573 (565, 581)	269 (263, 276)	62 (58, 65)
Obese	232	630 (620, 641)	230 (222, 238)	44 (40, 48)
*p*-value		< 0.001	< 0.001	< 0.001
Self-rated health				
Excellent	202	538 (527, 550)	290 (280, 300)	75 (70, 81)
Very good	450	566 (558, 574)	271 (264, 277)	68 (64, 71)
Good	315	581 (569, 592)	269 (260, 278)	54 (51, 58)
Fair	68	604 (583, 625)	257 (239, 275)	43 (37, 49)
Poor	9	615 (564, 666)	260 (211, 310)	29 (5, 53)
*p*-value		< 0.001	0.002	< 0.001
Depressive symptoms				
CESD score < 10	959	568 (562, 574)	273 (268, 278)	63 (60, 65)
CESD score ≥ 10	85	594 (575, 614)	261 (244, 278)	49 (42, 56)
*p*-value		0.014	0.183	< 0.001
Cognitive impairment				
No	1018	572 (567, 578)	270 (266, 275)	61 (59, 64)
Yes	26	550 (506, 593)	294 (257, 330)	60 (51, 70)
*p*-value		0.320	0.222	0.836
Difficulty walking half mile				
None	797	554 (547, 560)	280 (274, 285)	71 (68, 73)
Some	145	586 (572, 600)	271 (259, 283)	47 (42, 52)
A lot / Unable	102	629 (609, 650)	239 (222, 257)	35 (30, 41)
*p*-value		< 0.001	< 0.001	< 0.001
Hypertension				
No	562	560 (551, 569)	276 (269, 283)	68 (64, 71)
Yes	482	581 (574, 589)	267 (261, 274)	55 (52, 58)
*p*-value		0.001	0.067	< 0.001
Coronary artery disease				
No	908	566 (559, 572)	275 (269, 280)	64 (61, 66)
Yes	136	599 (584, 614)	255 (243, 267)	50 (44, 56)
*p*-value		< 0.001	0.004	< 0.001

^a^ Values shown in the table are the means (95% confidence intervals) of the average daily pattern metric adjusted for wear time and demographic factors (age, gender, race/ethnicity, and education) and weighted to account for selection due to factors related to device-wear consent. *P*-values correspond to tests for differences in means across levels of the factors. The selection model used to generate weights was based on all covariates shown in Table 1. ActiGraph-wear participants missing covariates that were used in the selection model (*N* = 44 participants) were excluded from the above weighted summary table

^b^PA classified based on OPACH cut-points [25]: Sedentary Time (VM < = 18 counts/15 s); LPA (VM is 19 through 518 counts/15 s); and MVPA (VM is > 518 counts/15 s)

## Discussion

In the ACT-AM study, it was feasible to recruit an older population to wear two activity monitoring devices. Our overall recruitment rate (~ 60%) is very similar to the rates observed in a prior study [40] that recruited older men from the British Regional Heart Study in which 55.5% agreed to wear an ActiGraph device. In ACT-AM, those that consented to wear the devices were younger and healthier (lower prevalence of various health conditions) than those not consenting. The main reason for declining to wear the devices was not being interested. There could have been some embarrassment and reactivity associated with being asked to wear the devices, particularly for those experiencing health declines.

Across the board, sedentary time was higher and physical activity lower among those who were older and who had chronic conditions and/or difficulty walking. Accounting for differential consent using inverse probability weighting, we found that older adults engage in high levels of sitting time when posture-specific devices were used, overall an average of 10.2 h per day. This is one of the first studies to examine sitting patterns among the oldest old; indeed, participants aged 90+ sat for 11.6 h per day on average. Our estimates are higher when compared to other studies of older adults. The estimated prevalence of sedentary behavior pooled across 22 studies in older adults was 9.4 h per day (mean age was 72) according to a review paper [41]. Sedentary time ranged from 8.5 to 10.7 h per day and studies with older mean ages reported higher sedentary time. None of these studies used estimates from posture-specific devices like activPAL [11]. There are a few recent studies using activPAL in older populations that were not included in the review [34, 42, 43]. In the Maastricht Study, middle-aged and older participants without metabolic conditions sat for 9.1 h/day while those with metabolic conditions spent 10.1 h/day sitting [42]. In the AusDiab study, adults age 75 and over sat for 9.2 h per day on average [43]. In the Interactive Diet and Activity Tracking in AARP (iDATA) study [34], adults aged 50–74 sat for 9.8 h per day on average. Our results indicate estimates on the higher end of what has been observed in the literature; this could be attributed to our population which included very old adults and people with various health conditions [41, 44] and to our study’s ability to correct for selection bias due to consent, which most other studies cannot do. Indeed, there have been few published studies that included adults over age 90 so our finding that this age group has the highest levels of sitting time add more information to the literature base [41].

We examined several other metrics of sitting time including mean bout duration and number of prolonged sitting bouts. Longer mean bout length has been associated with developing metabolic syndrome in working age individuals [45], cardiovascular disease in older women [46], and reduced physical function in older adults [47, 48] so could be a particularly important metric. We found that participants over age 90, with obesity, difficulty walking, and chronic conditions had longer mean bout length and more prolonged sitting bouts. We also observed that men had longer mean bout length and more 30 min or longer bouts than women. In the Maastricht Study [33], the sample engaged in about 4.8 prolonged sedentary bouts per day (our average was 5.9) and daily mean bout duration was about 11 min per bout (ours was 17 min per bout). We did not observe as much variability across demographics and health conditions for breaks from sitting, however this metric may be less helpful because people who sit less also engage in fewer breaks from sitting. For this reason, examining prolonged bouts and mean bout duration make more sense for interpretation. In the Maastricht study, breaks from sitting were lower among people with obesity (about 51 breaks per day) compared to non-obese participants (about 55–56 breaks per day). In our study, normal weight older adults engaged in an average of 46 breaks per day compared to 38 among those with obesity. In ACT-AM breaks were lower than what was observed in the Maastricht Study, however the mean age in that study is younger at 60 years compared to our sample (77 years in the ACT-AM sample).

Moderate-to-vigorous physical activity among the ACT cohort was approximately an hour per day based on ActiGraph based measures. This was higher than in other studies, though direct comparisons are hard to interpret because studies may use different cut-points to determine MVPA. Indeed, many accelerometer studies in older populations use cut-points derived in younger populations. The Women’s Health Initiative OPACH study included a calibration study and derived cut-points that can be used more accurately in older adults [25], and those cut-points were utilized for our analysis. Similarly, in that study, older women had 50 min of MVPA on average per day [49]. For comparison purposes, processing the ACT cohort ActiGraph data according to the Troiano cutpoint (2020 counts per minute) [50], we derived a mean of 14 min per day of MVPA. In the National Health and Nutrition Examination Study (NHANES), older adults engaged in 42 min of MVPA per day (using a cut-point of 1000; 10.8 min/day when using a cut-point of 2000 counts per minute) [44]. Our estimates for MVPA are, therefore, similar to those found in other cohorts. Overall, the ACT-AM sample engaged in high levels of physical activity and also spent the vast majority of their day sitting, indicating high levels of sedentary behavior.

We measured sedentary time in various ways for the ACT-AM sample. ActiGraph measures movement and its metric of sedentary time is actually a measure of non-movement (better termed inactivity). The activPAL measures posture and thus can differentiate sitting from standing postures. The two are essentially measuring different aspects of sedentary behavior. Measured by ActiGraph, participants in ACT-AM engaged in 9.5 h of sedentary time or inactivity per day on average. Measured by activPAL, participants in ACT-AM engaged in 10.2 h per day of sitting time. In the future, we will be able to use the ACT-AM data to examine differences in measurement of sedentary time between the devices.

The ACT-AM study has provided a tremendous opportunity to collect novel data on how patterns of sedentary and active time are related to cognitive and physical health in older adults, and ultimately these cohort-based data can be leveraged to investigate factors associated with resilience in aging. In particular, the use of activPAL will allow us to determine whether small daily differences in posture could relate to important age-related outcomes. For example, recent research suggests that those who take more regular breaks from sitting have better physical function [47, 48]. Research has also demonstrated that sedentary behavior, particularly in those over age 80 [51] and with functional limitations [52] is associated with fall risk. Research is beginning to describe some associations between sedentary time and cognitive function. For example, a recent longitudinal study found that increased sedentary time, as measured via ActiGraph, was associated with worsened cognitive ability over nearly 2 years of follow-up [53]. Another recent study showed that higher sedentary time was associated with lower brain-derived neurotrophic factor levels [54]. A recent lab study suggested that uninterrupted sitting reduced cerebral blood flow which was offset with 2-min walking breaks every 30 min [55]. Eventually the ACT-AM Study data can be used to link sedentary and physical activities to cognitive and physical function as well as morbidity and mortality.

A strength of our study is the inclusion of people who are among the oldest old and that had a variety of health conditions who are retained, in part, because of ACT’s ability to conduct assessment visits in the home. ACT also captures detailed health information across a wide variety of domains. Including two devices to more accurately measure both sedentary behavior and physical activity is another study strength. Finally, we were able to use weighting to account for factors related to device-wear consent so that our summaries of the pattern data better generalize to the ACT cohort population. Limitations include a study population that is largely educated and white (reflecting local demographics), limiting broader generalizability.

## Conclusion

It was feasible to collect activPAL and ActiGraph data within a community-based cohort of older adults. The data being collected in this ACT sub-study can help better elucidate the relationships between patterns of sitting and physical activity and important aging-related outcomes such as cognitive and physical function over time in future investigations.

## Data Availability

The datasets used and/or analyzed during the current study are available from the corresponding author on reasonable request.

## Abbreviations

ACT: Adult Changes in Thought
ACT-AM: Adult Changes in Thought – Activity Monitor
VM: Vector Magnitude
SD: Standard Deviation
KPWA: Kaiser Permanente Washington
DSM-IV: Diagnostic and Statistical Manual of Mental Disorders, Fourth Edition
EHR: Electronic Health Records
iDATA study: Interactive Diet and Activity Tracking study
BMI: Body Mass Index
CES-D: Center for Epidemiologic Studies Depression Scale
CASI: Cognitive Abilities Screening Instrument
NHANES: National Health and Nutrition Examination Study
OPACH: Objective Physical Activity and Cardiovascular Disease Health in Older Women Study
MVPA: moderate to vigorous physical activity
